# Origin and early development of the chicken adenohypophysis

**DOI:** 10.3389/fnana.2015.00007

**Published:** 2015-02-17

**Authors:** Luisa Sánchez-Arrones, José L. Ferrán, Matías Hidalgo-Sanchez, Luis Puelles

**Affiliations:** ^1^Faculty of Medicine, Department of Human Anatomy, School of Medicine and IMIB (Instiuto Murciano de Investigación Biosanitaria), University of MurciaMurcia, Spain; ^2^Department of Cell Biology, Faculty of Science, University of ExtremaduraBadajoz, Spain

**Keywords:** adenohypophysis, anterior pituitary, fate map, pre-placodal ectoderm, placodes, gene markers, Rathke’s pouch

## Abstract

The adenohypophysis (ADH) is an important endocrine organ involved in the regulation of many physiological processes. The late morphogenesis of this organ at neural tube stages is well known: the epithelial ADH primordium is recognized as an invagination of the stomodeal roof (Rathke’s pouch), whose walls later thicken and differentiate as the primordium becomes pediculated, and then fully separated from the stomodeum. The primordium attaches to the pial surface of the basal hypothalamus, next to the neurohypophyseal field (NH; future posterior pituitary), from which it was previously separated by migrating prechordal plate (pp) cells. Once the NH evaginates, the ADH surrounds it and jointly forms with it the pituitary gland. In contrast, little is known about the precise origin of the ADH precursors at neural plate stages and how the primordium reaches the stomodeum. For that reason, we produced in the chicken a specific ADH fate map at early neural plate stages, which was amplified with gene markers. By means of experiments labeling the mapped presumptive ADH, we were able to follow the initial anlage into its transformation into Rathke’s pouch. The ADH origin was corroborated to be strictly extraneural, i.e., to lie at stage HH4/5 outside of the anterior neural plate (anp) within the pre-placodal field. The ADH primordium is fully segregated from the anterior neural border cells and the neighboring olfactory placodes both in terms of precursor cells and molecular profile from head fold stages onwards. The placode becomes visible as a molecularly characteristic ectodermal thickening from stage HH10 onwards. The onset of ADH genoarchitectonic regionalization into intermediate and anterior lobes occurs at closed neural tube stages.

## Introduction

The pituitary gland of vertebrates is a key regulator of the endocrine system, required for the maintenance of reproduction, growth, homeostasis and metabolism. It develops by apposition of two embryonic primordia: the posterior pituitary gland, or neurohypophysis (NH), which is a dock for release of the hypothalamic neurohormones vasopressin and oxytocin are into the bloodstream, and the anterior pituitary gland, or adenohypophysis (ADH), which represents a major endocrine control organ (Herzog et al., 2004; Guner et al., 2008). The NH develops as a digitiform median evagination from the hypothalamic tuberal neuroepithelium, at the area known as “median eminence” (Cobos et al., 2001; Puelles and Rubenstein, 2003; Herzog et al., 2004).

The ADH origin is still under discussion, since different developmental scenarios have been conjectured in the light of fate-mapping experiments in diverse vertebrates. The controversy centers on whether the ADH origin is neural or non-neural. Various fate-mapping studies performed at neurula stages in zebrafish, frog, and chick embryos reported that the ADH arises at the rostral border of the neural plate (known as the “anterior neural ridge”, ANR; Takor and Pearse, 1975; Couly and Le Douarin, 1985; Eagleson et al., 1986; Couly and Le Douarin, 1987; elAmraoui and Dubois, 1993; Dubois and Elamraoui, 1995; Whitlock and Westerfield, 2000; Whitlock et al., 2003; Herzog et al., 2004). Specifically, Couly and Le Douarin (1988) thought that this early neural ADH primordium was continuous with the presumptive hypothalamic NH primordium (possibly suggesting, but not reasoning out, an implicit answer to the question about why ADH and NH later come to be joined in the pituitary gland).

In contrast, other experimental work done in mammals and birds suggested that the primary ADH domain is not neural and arises from a median placodal ectodermal domain placed in front of (outside) the ANR. For instance, Cobos et al. (2001) concluded that the avian ANR domain proper contains exclusively prospective telencephalic cells (never giving rise to NH or ADH cells when grafted selectively). The ADH primordium was labeled only when the ectoderm forming the outer non-neural slope of the ANR was included in the grafts. This distinction was not made by earlier authors reporting such experiments. These results implied that the non-neural ADH primordium is separated from the prospective tuberal NH by several interposed neural domains, such as the telencephalic preoptic area (later represented at the midline by the lamina terminalis) and the hypothalamic chiasmatic, retrochiasmatic and tuberal areas. Their subsequent meeting must be due to parallel morphogenetic changes in position of the ADH and NH primordia. These results about the strictly telencephalic character of the ANR neural plate domain were corroborated later by more recent fate maps of the chicken neural plate (Fernández-Garre et al., 2002; Sanchez-Arrones et al., 2009, 2012; Cajal et al., 2012, 2014); see also zebrafish and frog fate maps (Houart et al., 1998; Eagleson and Dempewolf, 2002).

Everybody in the field agrees that Rathke’s pouch, a dorsal evagination of the stomodeal roof, represents the immature ADH later in development (Cobos et al., 2001; Rizzoti and Lovell-Badge, 2005). It is also widely accepted that the stomodeal roof is ectodermal in character, rather than endodermal, thus excluding the possibility of an endodermal origin of the ADH. Nevertheless, it was found that the foregut endoderm underlying the early ANR is required during the specification of both the anterior neural border and the ADH primordium (Withington et al., 2001; Sanchez-Arrones et al., 2012).

There is little information, though, about how the early ADH primordium, be it neural or non-neural, comes to occupy the position of Rathke’s pouch under the hypothalamus. This suggests the need of a detailed fate map and morphogenetic follow-up at several early developmental stages, in order to understand more fully the early development of the anterior pituitary gland (Figure 7). In this essay we re-examined in detail the origin and subsequent changing position of the ADH epithelial plate with regard to neighboring tissues at various stages. To address this issue, we first examined the early ADH field by fate mapping at neural plate stages (HH4/5) and survival up to closed neural tube stages (HH12), using the chick embryo as a model system. These data showed that the ADH precursors are located within an area distant 260–290 μm from the node in the median non-neural ectoderm rostral (one might rather say “dorsal”, or “peripheral”) to the ANR. This ADH primordium is a close neighbor of the rostral midline telencephalic domain (prospective subpallial cells; see Cobos et al., 2001; Sanchez-Arrones et al., 2009; Cajal et al., 2014); the prospective ADH ectoderm was thus again found to lie just outside the neural plate and quite distant from the prospective NH. Moreover, the ADH primordium directly overlies the boundary between the extraembryonic hypoblast and the rostralmost foregut endoderm precursors (Kimura et al., 2006).

**Figure 1:**
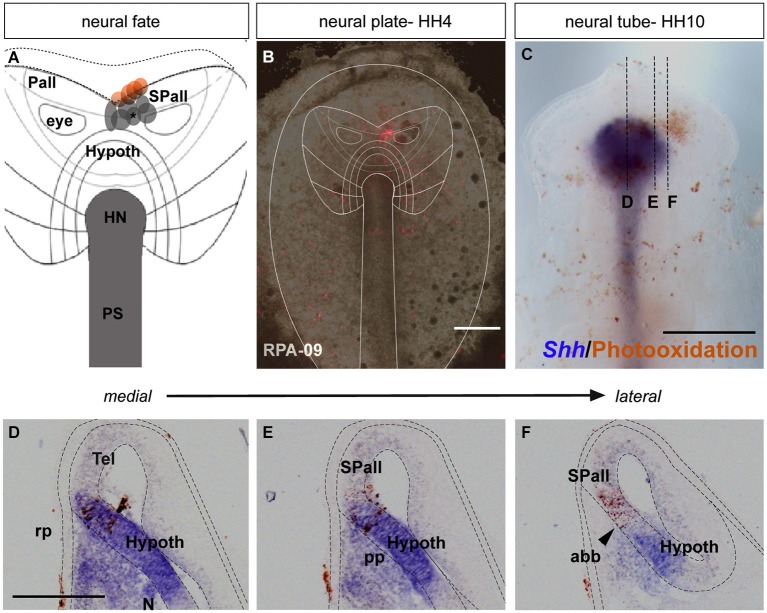
**Neural fate: analysis of the representative experimental case rostral plate area (RPA)-09. (A)** Map of all studied grafts or injections corresponding with a neural fate. Those marked in red also labeled partially the anterior neural border and/or the ADH field. **(B)** The immediate bright field whole-mount view of case RPA-09 shows that the DiI injection was located within the neural plate, ranging between the prospective preoptic telencephalon and the more ventral basal hypothalamus. **(C)** At stage HH10, the embryo was whole-mount- hybridized for *Shh* (to visualize the hypothalamic basal plate expression), and the DiI was photo-oxidated with DAB. **(D–F)** Sagittal cryo-sections show that most labeled cells extend dorsoventrally within the rostral neural tube. In mid-sagittal sections, the injection-derived cells partially overlap the *Shh* positive cells of the hypothalamic basal plate. Laterally, positive cells appear in alar hypothalamic and telencephalic domains, where *Shh* expression is absent. Scale bars: 250 μm in **(B,C)**; 125 μm in **(D–F)**.

**Figure 2:**
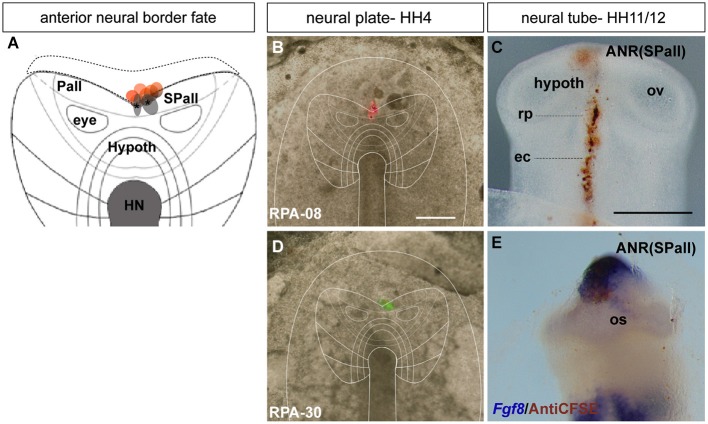
**Anterior neural border fate: analysis of the experimental cases RPA-08 and RPA-30. (A)** Map of studied grafts or injections correlative with an anterior neural border fate. The grafts marked in red also produced ADH labeling. **(B,D)** Cases RPA-08 and RPA-30 in bright-field whole-mount view, illustrating an injection and a graft, respectively, targeting the previously fate-mapped rostral neural boundary. **(C)** At stage HH11–12, the DiI-labeled RPA-08 cells were found in a median elongated strip extending across the neural/non-neural midline, with part of the labeled cells within the telencephalic domain and another part extending into the prospective Rathke’s pouch and median head ectoderm approaching the anterior intestinal portal. The graft-derived cells largely were located at stage HH12 laterally at the prospective subpallium and ventrally at the prospective optic stalk. **(E)** In case RPA-30 some labeled cells overlap with *Fgf8* expression in the neural and non-neural ectodermal cells. Scale bars: 250μm.

**Figure 3:**
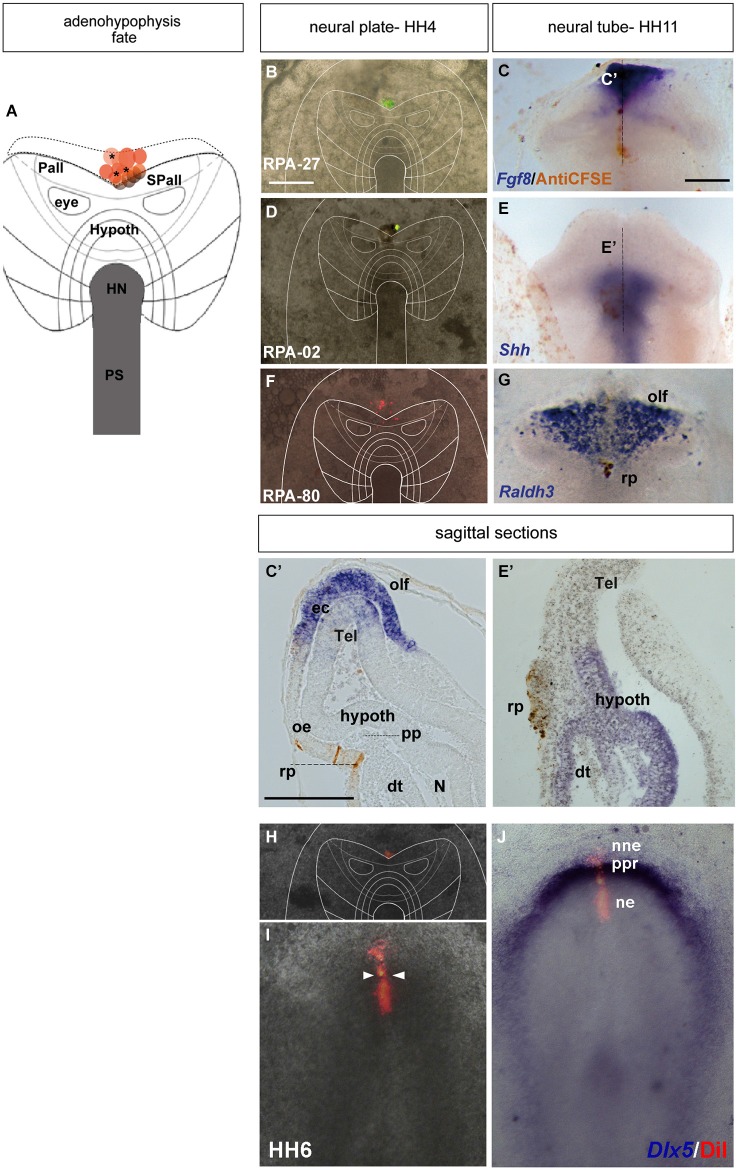
**Adenohypophyseal fate: analysis of the experimental cases RPA-27, RPA-02 and RPA-80. Initial median cell movements. (A)** Map of all studied grafts or injections corresponding with partial adenohypophyseal fate. **(B,D,F,H)** Combined fluorescent and bright field images of representative HH4 chick embryos in which a graft **(B)** or either a DiI **(D)** or DiO injection** (F,H**) was placed at the median non-neural ectodermal region. **(C,C’)** At stage HH11, the graft-derived cells were located in the rostromedian non-neural ectoderm, largely coinciding with the *Fgf8*-negative Rathke’s pouch rudiment. **(E,E’)** The prospective Rathke’s pouch tissue, flipped over into external contact with the neural terminal wall, already has elongated to the level of the *Shh*-positive prospective hypothalamic basal plate. **(F,G)** In case RPA-80, the labeled non-neural cells of the ADH primordium clearly appeared at the medial head ectoderm, within the space that separates the bilateral *Raldh3*-positive olfactory placodes, whose cells remained unlabeled. **(I,J)** Simple fluorescent and combined fluorescent and bright field images of the case illustrated in **(H)**, counterstained with *Dlx5* whole-mount ISH, showing the elongated median labeled trace of ectodermal tissue extending from the preplacodal field (ppf) into the ventrally displaced ADH primordium, at stage HH6. Arrowheads in **(I)** mark the site of the original injection (see also Sanchez-Arrones et al., 2012). Scale bar: 250 μm in **(B,C)**; 125 μm in **(C’)**.

**Figure 4:**
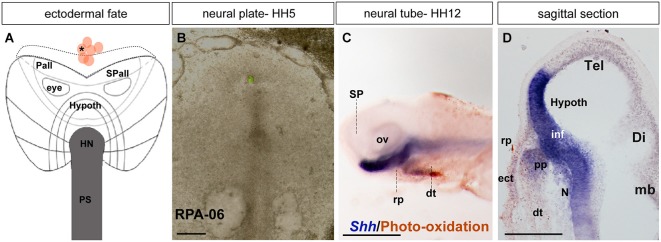
**Ectodermal fate: analysis of the experimental case RPA-06. (A)** Map of studied grafts or injections exclusively corresponding to non-neural head ectoderm at the midline. **(B)** Bright-field image of representative case RPA-06 illustrating labeled cells (corresponding to dark dot in **A**) located largely in the rostral ectoderm beyond the ADH field locus. **(C)** Side view of the whole-mount HH12 specimen after photo-oxidation of the DiI and hybridization for *Shh* (neural basal and floor plate); DiI labeled cells appear in the median head ectoderm and slightly extend into the digestive tube. **(D)** A mid-sagittal section through the specimen shows that the brown-stained labeled cells lie outside the Rathke’s pouch primordium (rp). Scale bars: 250 μm in **(B)**; 125 μm in **(D)**.

**Figure 5:**
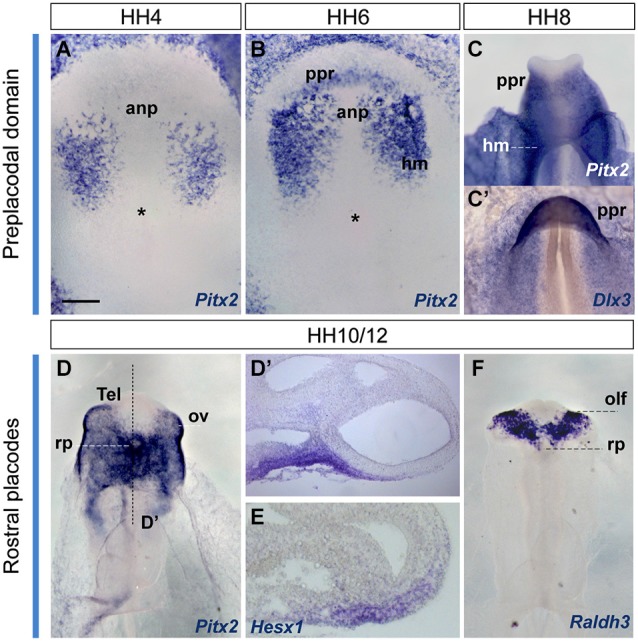
**Early gene expression at the ADH primordium**. Neural plate and neural tube whole mounts hybridized with *Pitx2*, *Dlx3*, *Hesx1* and *Raldh3*. The images are shown in a ventral view. The node is marked by an asterisk in some of the images. Scale bar: 250μm.

**Figure 6:**
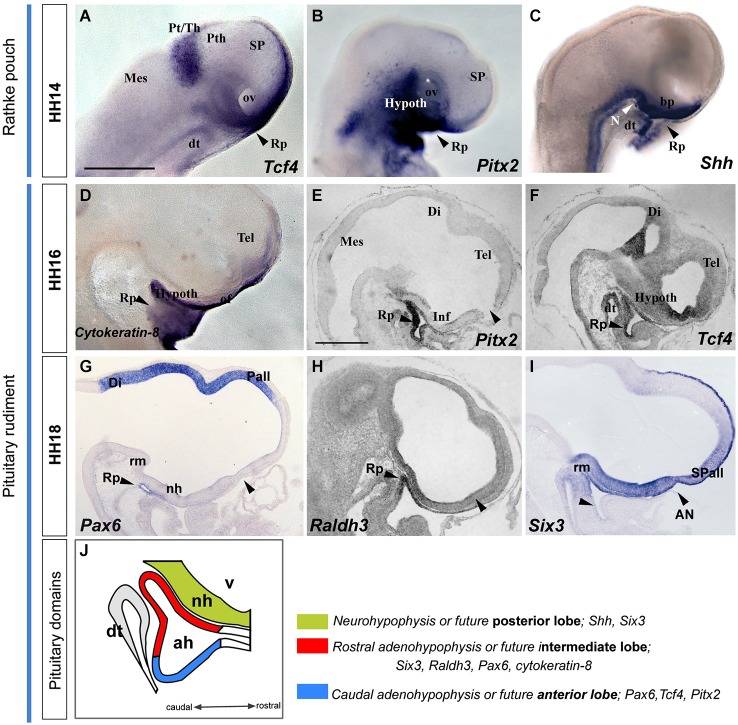
**Latter molecular expression at the ADH and NH primordium, pituitary rudiment**. This panel shows the expression pattern of some genes involved in ADH and NH development. **(A–I)** Whole-mount hybridized embryos for* Tcf4, Pitx2, Shh*, *Cytoqueratin-8, Pax6, Raldh3* and *Six3*. **(J)** The schema represents the pituitary rudiment and its initial molecular subdivision. Scale bar: 250μm.

**Figure 7:**
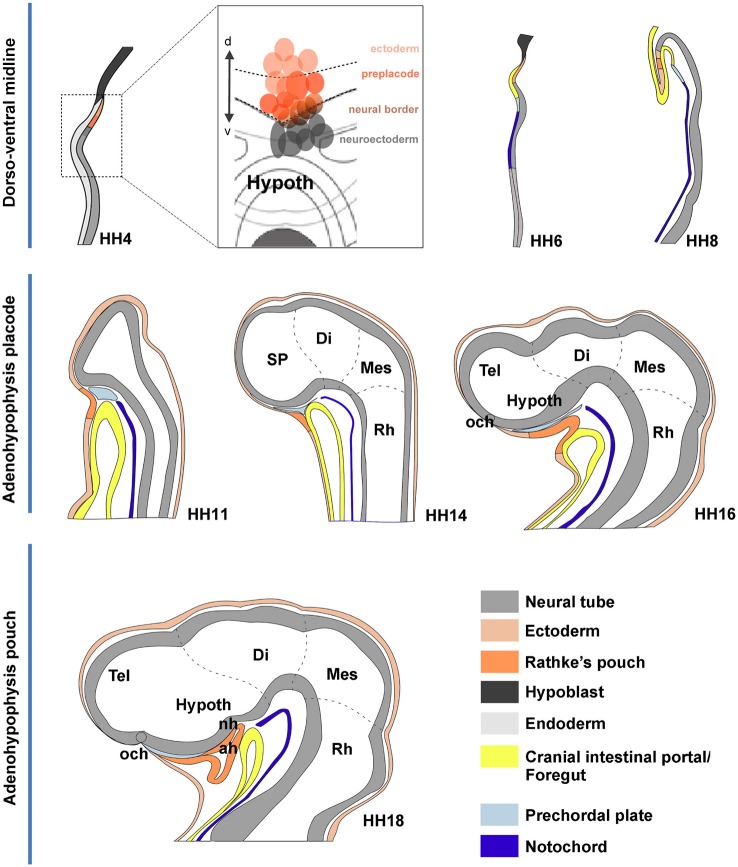
**Schematic summary of proposed morphogenetic events leading to the development of the adenohypophysis across neural plate and neural tube stages**. These schemata each represent in general the changing sagittal midline of chick embryos at different developmental stages. The dashed lines delimit the main rostrocaudal subdivisions of the neural tube. **(HH4)** The initial schema illustrates a median section through the early neural plate, marking tissue corresponding to hypoblast (black), rostral endoderm (pale gray) and neural plate or tube (gray). The prospective ADH, representing median non-neural ectoderm, is coded in deep orange. The deduced flat fate map of the median anterior head domain is illustrated in the insert at the side, showing in deep red the labeled sites identifying by fate the ADH field, in contrast to rostral neuroectoderm sites (gray field) and non-ADH head ectoderm sites (light red). **(HH6)** This schema is similar to the HH4 one, though we distinguish now the endodermal primordium of the anterior intestinal portal (yellow) and the notochordal tissue (deep blue). **(HH8)** The head fold develops, causing the ADH field to flip over in a hinge-like motion under the anterior intestinal portal, though it still remains transiently attached by head ectoderm to the anterior neural ridge (rostral neuropore still open). **(HH11)** Neurulation is nearly finished at this stage; we identify the head ectoderm (weak orange) and the prechordal plate mesoderm (weak blue) at the tip of the notochord (the latter lies under the prospective mammillary pouch); note the prechordal tissue transiently separates the ADH rudiment from the basal hypothalamus. **(HH14,HH16)** The prechordal tissue gradually migrates dorsalward in front of the terminal hypothalamic wall, allowing the stomodeum to form and the ADH to approach the hypothalamus. **(HH18)** The prechordal cells have migrated away, being now in contact with the alar median hypothalamus (chiasmatic region) and the prospective preoptic telencephalon (terminal lamina); they leave the basal tuberal hypothalamus free for close contact with the ADH primordium; this rostroventral tuberal territory includes the prospective neurohypophysis (NH), whose evagination is first observable several stages later, at HH26.

## Materials and methods

### Fate map of the rostral midline ectoderm

Fertilized chicken eggs were incubated at 38°C to reach the desired stage. Fate-mapping experiments (Tables 1, 2) were performed at stage HH4/5 (Hamburger and Hamilton, 1951), with focal injections (Selleck and Stern, 1991) of carbocyanine dyes DiI (1, 1′-dioctadecyl-3,3,3′,3′-tetramethylin-docarbocyanine perchlorate; Invitrogen, D282) and/or DiO (3,3′-dioctadecyloxacarbocyanine perchlorate; Invitrogen, D275) into the rostromedian neural and non-neural ectoderm in New-cultured chick embryos (New, 1955; protocol modified by Stern and Bachvarova, 1997). The injection was performed by applying buccal pressure to tubing connected with glass micropipettes filled with the dye solution. In some cases, homotopic grafts labeled with green fluorescent 5-(and-6)-carboxyfluorescein diacetate, succinimidyl ester (CFSE) (Molecular Probes) were performed as detailed by Fernández-Garre et al. (2002). The operated embryos were recorded photographically under fluorescent illumination using an Axiocam camera (Carl Zeiss Vision; München-Hallbergmoos), immediately after the graft or injection. The graft was detected by the green/red (DiI/DiO) fluorescent signal of the carbocyanines, respectively (Hatada and Stern, 1994). The embryos were cultured further until they reached stages HH10–14 and were then fixed overnight in cold 4% paraformaldehyde (phosphate buffered pH 7.4, 0.1M, 4°C).

**Table 1:** **Measurements of the experimental cases, data taken at HH4/5**.

Case	Stage at transplantation	Stage of fixation	Angle	Distance from HN (μm)	Diameter of the graft or injection (μm)	Distance (μm)
RPA-02	HH4	HH11+	0–5°	274	77.4	351.4
RPA-03	HH4	HH10	5–10°	268	60	328
RPA-05	HH4	HH10+	0–5°	262	113	375
RPA-25	HH4	HH10+	0–5°	280	65.5	345.5
RPA-27	HH4	HH11+	0–5°	260	48	308
RPA-81	HH4	HH11	0–5°	310	84	394
RPA-80	HH4	HH10	0–5°	245	150	395
RPA-08	HH4	HH11	0–5°	173	113	286
RPA-06	HH5	HH12	0–5°	280	37.5	317.5
RPA-22	HH4	HH10	0–5°	234	84	318
RPA-30	HH4	HH12	0–5°	217	56	273
RPA-09	HH4	HH10	5–10°	170	42.5	212.7
RPA-26	HH4+	HH12	0–5°
RPA-10	HH4	HH9+	15–20°	333	61	394
RPA-12	HH4	HH9	0–5°	350	67	417
RPA-18	HH4	HH11	5–10°	384	56	440
RPA-84	HH4	HH9+	10–15°	333	45	378
RPA-88	HH4	HH10	20–40°	417	84	501
RPA-11	HH4+	HH11+	0–5°
RPA-29	HH4	HH9	15–20°	213	58	271
RPA-20	HH4	HH11	15–20°	223	70	293
RPA-14	HH4	HH11	0–5°	186	64	250
RPA-87	HH4	HH11	10–25°	245	27	272

**Table 2:** **Correlation of medio-lateral and dorso-ventral extent of derived graft domains with boundaries of gene expressions in the extra and neural areas**.

				Ectoderm			Secondary prosencephalon		
Case	Gene expression	Fixation stage	Endod	Rostral	Medial	Lateral	ANB	SPall	Hypoth: os
RPA-02	Shh	HH11+	+	+	+++
RPA-03	Shh	HH10	+	+++	++			++	
RPA-05	Shh	HH10+	+	+++			+	+	
RPA-25	Fgf8	HH10+		++
RPA-27	Fgf8	HH11+		++	+++				
RPA-81	Raldh3	HH11		++	+++
RPA-80	Raldh3	HH10		+	+++
RPA-08	-	HH11		+++	+++		++	++
RPA-06	Shh	HH12			+++
RPA-22	Fgf8	HH10		++			++	+
RPA-30	Fgf8	HH12		+			++	++
RPA-09	Shh	HH10					+?	++	++
RPA-26	Fgf8	HH12		++		+	+	+	
RPA-10	Shh	HH9+		+		++
RPA-12	Fgf8	HH9		+		++
RPA-18	Fgf8	HH11				+++
RPA-84	-	HH9+				+++
RPA-88	-	HH10				++
RPA-11	Fgf8	HH11+					+	+
RPA-29	Fgf8	HH9						++	+
RPA-20	Fgf8	HH11						++	+
RPA-14	Fgf8	HH11						++	+
RPA-87	-	HH11						+++	+++

*The relative amount of labeled cells filling the respective AP (medial-lateral) or DV (dorsoventral) territories is estimated roughly as “few cells” (+), “up to half the domain is labeled” (++) and “most or all the domain is labeled” (+++)*.

### Photo-oxidation of carbocyanine-dye-labeled cells

To visualize permanently the dye-labeled cells, the fluorescence was photo-converted to an insoluble diaminobenzidine (DAB) by photo-oxidation (described by Selleck and Stern, 1991). The embryos were removed from the PFA and incubated with DAB solution (DAB in 0.1M Tris pH 7.4) in the dark, at room temperature, for 1 h. Each specimen was placed in a fairly deep glass cavity slide, covered with a coverslip. Under microscope epifluorescence the regions containing labeled cells were illuminated until all fluorescence disappears. After that, most operated specimens were systematically processed for *in situ* hybridization (ISH) with one of the mRNA probes described below.

### *In situ* hybridization (ISH)

The embryos were hybridized following the protocol described by Streit and Stern (2001), Ferran et al. (in press). For this study we normally used digoxigenin-uridin triphosphate (UTP)-labeled antisense chicken riboprobes. The standard visualization procedure with nitro blue tetrazolium (NBT)/5-bromo-4-chloro-3′-indolyphosphate p-toluidine salt (BCIP) solution as chromogenic alkaline phosphatase substrate gave a dark blue reaction product for digoxigenin-UTP. The gene markers analyzed were *Pitx2*, *Shh*, *Fgf8*, *Raldh3*, *Tcf4*, *Cytoqueratin-8*, *Hesx1, Dlx3, Pax6* and *Six3*.

### Immunohistochemitry (IHC) and tissue processing

Cells derived from the CFSE-labeled grafts were visualized after the ISH reaction. The embryos were immunoreacted with anti-fluorescein Fab fragments conjugated to horseradish peroxidase (anti-fluorescein-peroxidase, horseradish (POD); 1:500; Boehringer; Mannheim) following standard protocols (Fernández-Garre et al., 2002; Ferran et al., in press). Afterwards the embryos were cryoprotected overnight in 10% sucrose in PBS, and embedded in 10% gelatin and 10% sucrose in PBS. The blocks were cryostat-sectioned 10–12 μm-thick in the sagittal plane and mounted with Mowiol.

### Imaging

Images were captured with an Axiocam digital camera (Carl Zeiss Vision; München-Hallbergmoos). Digital images were processed with Photoshop® CS4 11.0.2 and ImageJ (Fiji) software. Representative images were used to draw vectorial schemata with Adobe Illustrator CS4.

As previously described (Streit, 2002), embryos subjected to ISH shrink significantly when heated in the presence of formamide and detergent. Two approaches were used to overcome this problem when comparing the position of DiI-labeled cells. In some embryos the images obtained from *in situ* hybridized embryos were increased by 10% and aligned to landmarks on the fluorescence image to produce a montage. In other embryos, the DiI fluorescence was photoconverted with DAB (Izpisúa-Belmonte et al., 1993) before ISH. This approach allows DiI labeled cells to be seen directly in the whole-mount *in situ* processed embryo and confirms the assumptions made by adjusting the magnification of separately obtained images, as described above (Sanchez-Arrones et al., 2012).

## Results and discussion

### Experimental fate mapping and early morphogenetic tracing of the prospective ADH

During chick development, the ADH placode (presumptive Rathke’s pouch) becomes morphologically identifiable as a thickened patch of epithelium at stages HH12–14 (Romanoff, 1960). At this moment the ADH is molecularly segregated from the adjacent olfactory placode, both being ectodermal placodal specializations that lie outside the neural tube. In order to know precisely the position of the prospective ADH primordium relative to the neural telencephalic and neurohypophyseal primordia at earlier neural plate stages, when the ADH is not histologically distinct, we performed fate-mapping experiments combined with mappings of neural and non-neural maker genes. To this end, the anterior midline ectoderm was labeled systematically using small fluorescent grafts or small single injections of the fluorescent dyes DiI or DiO at stage HH4/5 at different dorsoventral levels (the node represents the ventralmost median position and the median non-neural ectoderm lies topologically dorsal to the ANR (Fernández-Garre et al., 2002; Sanchez-Arrones et al., 2009). The derived domains of these grafts and injections were examined in comparison to a selected gene marker between stages HH10 and HH12, when the placodal cells start to be morphologically distinguishable (thickened and invaginated) from the adjacent ectoderm. The experimental cases selected for this analysis are illustrated in the Figures 1–4. These show schemata which respectively locate the set of grafts and injections grouped at the midline which produced derivatives at either the anterior *median neural domain* (preoptic telencephalon and hypothalamus; Figure 1), the anterior *neural border domain* (ANR; Figure 2), anterior median *pre-placodal domain* (ADH; Figure 3) and anterior non-placodal *ectoderm* and *endoderm* (Figure 4). The size, distance from the node, and angular radial position of the grafts and injections relative to the nodal median radius appears listed in Table 1. We will describe below six representative cases out of a total of 23, ordered according to their progressively more dorsal fates; neural fate (RPA-09), ANR (RPA-08 and RPA-30), anterior median pre-placodal domain (RPA-27, RPA-02 and RPA-80) and anterior non-placodal ectoderm/endoderm (RPA-06).

#### Anterior median neural cells

In the case RPA-09, a DiI injection was placed across the prospective alar/basal boundary of the anterior neural plate (anp), roughly 170 μm distant from the node (Figures 1A,B, compare (Sanchez-Arrones et al., 2009; Table 1). At stage HH10, labeled cells derived from the injection appeared precisely across the molecular limit separating the alar and basal plates (bp) of the secondary forebrain (Figure 1C). This specimen was processed to detect *Shh* signal, which represents a gene expressed selectively at this stage throughout the floor and bp of the secondary prosencephalon (SP; Bardet et al., 2010). Some *Shh*-positive cells overlapped with brown-labeled cells derived from the injection (implying the latter fell partially upon prospective basal hypothalamus), whereas other injection derivatives overlapped with more dorsal *Shh*-negative cells located in the median alar SP (prospective alar hypothalamus and/or preoptic subpallial telencephalon (Figures 1D–F; see Cobos et al., 2001; Bardet et al., 2010)).

#### Anterior neural border cells

In the case RPA-08, a DiI injection targeting the ANR was placed more dorsally across the border between neural and non-neural (preplacodal) domains at a distance of 286 μm from the node (Figures 2A,B; see Sanchez-Arrones et al., 2012). At stage HH11, the DiI-labeled cells were located at the midline of the prospective septal roof plate of the subpallial telencephalon and also formed a thin stripe extending along the midline of the ADH placode (Rathke’s pouch) and neighboring head ectoderm (Figure 2C). In other experimental embryos of this group, such as case RPA-30, in which a fluorescent graft was inserted at the rostral neural border approximately 273 μm distant from the node (Figures 2A,D), slightly laterally to the RPA-08 injection, the grafted cells appeared at stage HH12 (closed neural tube) exclusively at the prospective median telencephalon, represented by the *Fgf8*-expressing ANR area of the presumptive septum and the preoptic area, without labeling the ADH primordium (Figure 2E; see Cobos et al., 2001). Some labeled (grafted) cells were observed as well at the optic stalk (alar hypothalamus), which is also *Fgf8* positive (Figure 2E; Crossley et al., 2001). The positional similarity of the experiments producing both neural and placodal derivatives vs. those producing only neural derivatives suggests close vicinity between both domains across the neural/non-neural border.

#### Anterior preplacodal domain; prospective ADH precursors

The ADH precursors were labeled specifically in the next three cases: RPA-27 RPA-02 and RPA-80. In case RPA-27, a graft was placed more dorsally along the midline ectoderm, at a 260 μm distance from the node (Figure 3B), whereas in RPA-02 a DiI injection was introduced at 280 μm from the node (Figure 3D). In the whole-mounted embryos photographed at stage HH11, the graft-derived cells were found at the rostral midline ectoderm site which contains the thickened Rathke’s pouch (see Cajal et al., 2012); the latter and the surrounding rostral ectoderm do not express *Fgf8* (Figures 3C,C’). Rathke’s pouch, already occupying the stomodeal roof, lies below the prechordal plate (pp), which in its turn underlies the prospective hypothalamic bp (*Shh*-positive domain; Figures 3E,E’; García-Calero et al., 2008). The grafted/injected ADH areas showed intercalation of labeled cells with unlabeled ones, suggesting that a longitudinal intercalation mechanism is involved in the early morphogenesis of the ADH placode (see also Sanchez-Arrones et al., 2009). Laterally, further cells derived from the graft extended into the rostral head ectoderm (shown in the RPA-02; Figures 3E,E’), but not into the olfactory placode (Raldh3-positive cells; shown in the RPA-80 case; Figure 3G).

Consistently with this idea, we recently reported that a longitudinal median intercalation mechanism also obtains during cell movements of the anterior proneural zone at neural plate stages. In fact, the median cells are compactly stretched across the entire ectodermal midline, including the neural and non-neural territories (shown in Figures 3H–J; see also Sanchez-Arrones et al., 2012); this suggests that midline cells display a differential intercalative behavior compared with nearby more lateral (caudal) cells.

#### Anterior ectoderm and endoderm

In case RPA-06, a single DiO injection (green fluorescence) was placed at HH5 at the rostromedian non-neural ectoderm and underlying rostral endoderm (Figures 4A,B; see Stalsberg and DeHaan, 1968; Kimura et al., 2006). The embryo survived until HH12, and the resulting fluorescent-labeled cells were photo-oxidated, while the specimen was hybridized for *Shh* (Figure 4C). Most labeled cells appear located in or superficial to the rostral digestive tube (dt; Figure 4D. The *Shh* expression is visible at the prospective basal and floor plates of the SP (hypothalamus). Axial mesodermal tissues including the pp and the notochord are also positive for *Shh* (Withington et al., 2001; García-Calero et al., 2008). Some DiO-labeled cells were detected in the head ectoderm located in front of the pp (Figure 4D).

These results indicate that the fates of ectodermal cells occupying the rostral midline are well segregated at early neural plate stages. The ADH precursors occupy strictly extraneural positions (Figure 7). These cells separate the more dorsal midline ectoderm (prospective oral and head ectoderm) from the anterior neural border cells (ANR; prospective roof and median alar subpallial domains of the telencephalon). The latter territories are relatively distant from the prospective NH, located much more ventrally at neural plate stages, within the prospective basal hypothalamus. Once the neural tube is closed and the telencephalic vesicles start to emerge bilaterally, the ADH precursors restricted to the extra-neural midline (as well as the underlying rostral endoderm) are bent by the cephalic fold as by a hinge into the stomodeal roof. Both the preopto-hypothalamic acroterminal midline and the median cephalic ectoderm are stretched considerably by the growth of the head and associated cell intercalation phenomena (Sanchez-Arrones et al., 2009, 2012). The ADH primordium thus results separated from the median telencephalon and develops a new close relationship with the basal hypothalamus (prospective NH), thanks to the vacation of the space previously occupied by the prechordal mesoderm.

### Molecular tracing of adenohypophysial development

The development of the pituitary gland is a multistep morphogenetic process controlled by a genetic program (Sheng et al., 1997; Treier et al., 1998). Though we know various molecular traits involved in specification and differentiation of the different cell types of the ADH (Sheng and Westphal, 1999), little is known about the molecular patterns activated during the earliest stages of ADH development. Assuming a non-neural placodal nature of the ADH primordium (see results above, and references cited in the Introduction), our next aim was to examine the molecular signals that first specify differentially the rostral pre-placodal ectodermal region vs. the anterior neural ectoderm at neural plate stages; secondly, we wanted to study the initial regionalization of the presumptive pre-placodal territory into ADH vs. olfactory placodes at early neural tube stages, during the formation of Rathke’s pouch; finally, our attention centered on the onset of ADH molecular regionalization into the anterior and intermediate pituitary gland lobes.

#### Early ADH development: paraneural pre-placodal primordium from HH4 to HH12

In order to visualize the potential ADH primordium we mapped the *Pitx2* transcription factor. This gene is involved in head mesoderm patterning (Bothe et al., 2011), and in the establishment of embryonic left-right asymmetry and the fate of precardiac cells during cardiogenesis (García-Castro et al., 2000; Lopez-Sanchez et al., 2009). It also plays a key role during ADH development at neural tube stages, correlative with the emerging Rathke’s pouch (chick, Sjödal and Gunhaga, 2008; Parkinson et al., 2010; Xenopus, Schweickert et al., 2001; mouse, Drouin et al., 1998; Suh et al., 2002). Other *Pitx* family members as *Pitx1* and *Pitx3* were well characterized during early ADH development, at pre-placodal stages (mouse, Lanctôt et al., 1997; zebrafish, Dutta et al., 2005); however, the early *Pitx2* expression was not yet addressed. We thus examined *Pitx2* expression by ISH in whole-mount chick embryos and cross-sections, comparing with other genes known to be involved in pre-placodal/placodal specification, such as *Dlx3* (expressed in non-neural ectoderm; Dutta et al., 2005; Khudyakov and Bronner-Fraser, 2009), *Hesx1* (an ADH placode marker; Hermesz et al., 1996), and *Raldh3* (an olfactory placode marker; Sabado et al., 2012; Figure 5). Initially, at HH4, *Pitx2* signal was only present in the head mesenchyme (Figure 5A). Shortly afterwards, at HH6+/7-HH8, when the anterior neural border becomes sharp (Sanchez-Arrones et al., 2012), an ectodermal *Pitx2*-positive domain was detected in the rostral head fold, which coincides with the *Dlx3*-positive paraneural pre-placodal area (Figures 5B,C’); a similar result was reported during early placodal differentiation in mouse embryos and zebrafish larvae. At early closed neural tube stages, HH10-HH11, this paraneural *Pitx2* signal overlaps the expression domain of the placodal marker *Hesx1*, precisely in the thicker median placodal cells, which are held to represent the adenohypophyseal placode (Figures 5D,E; see also Sjödal and Gunhaga, 2008). *Raldh3*-positive cells were detected instead bilaterally in the prospective olfactory placode cells and the surrounding ectoderm, whereas *Raldh3* expression was completely absent at the ADH placode (Figure 5F). Accordingly, at stages HH10–11 the *Dlx3/Pitx2*-expressing primary paraneural pre-placodal ectodermal domain becomes regionalized in cellular and molecular terms, forming rostrally the median *Pitx2/Hesx1*-positive ADH placode and bilaterally the *Raldh3*-positive olfactory placodes (Bailey et al., 2006).

#### Later development of the ADH: Rathke pouch rudiment from HH14 to HH18

The developing pre-placodal domain subsequently undergoes changes in cell shape that drive the histologic differentiation of the placodes. The first change observed is a thickening of the placodal cells, which is generally accompanied by incipient invagination. The ADH placode starts to transform into a recognizable Rathke’s pouch during stage HH12 (Romanoff, 1960), when the neural tube is bending at the cephalic flexure. Parallel growth of the cephalic fold, with rostroventral protrusion of the embryonic head and intercalative elongation of the median preopto-opto-hypothalamic part of the neural plate, causes the oro-pharyngeal plate (originally formed rostral to the pre-placodal ectodermal band) to be internalized into the depth of the growing stomodeum. The median part of the placodal band sharply hinges under the closing rostral neuropore into the stomodeal roof, thus inverting its apico-basal orientation as it also undergoes intercalative elongation (Figure 7). The median stomodeal cells derived from the primary ADH placodal domain (prospective Rathke’s pouch) thus result placed underneath the tuberal hypothalamus (prospective neurohypophyseal primordium), though the two primordia are still mutually separated by the respective basal membranes and interposed prechordal mesoderm. Closure of the rostral neuropore and differential morphogenesis of the non-median tissues (mainly the evaginating telencephalic and eye vesicles, plus the olfactory placodes) increasingly separates the stomodeal ADH primordium from its earlier telencephalic neighbors (by stretching and growth of the interposed non-neural ectoderm), and the fused ANR transforms into the neural commissural septal plate (Puelles et al., 1987; Cobos et al., 2001). By stage HH14 the invaginated ADH primordium progressively forms the roof of Rathke’s pouch under the median tubero-infundibular forebrain area. From this stage onwards the ADH cells lie progressively closer to the presumptive NH (though the evaginated NH organ only emerges later, at stage HH26 in chick embryos), and molecular regionalization of the ADH primordium starts, as the stalk of Rathke’s pouch degenerates and disappears.

We illustrate in Figure 6 the correlative changes of some molecules involved in ADH and NH development, namely the markers with* Tcf4, Pitx2, Shh*, *Cytoqueratin-8, Pax6, Raldh3* and *Six3*. At HH14, the oral stomodeal ectoderm containing Rathke’s pouch is distinguished molecularly by selective expression of *Tcf4*, *Pitx2* and *Shh* (Figures 6A–C; Sjödal and Gunhaga, 2008). At HH16-18, the ADH pouch underlies the NH primordium and the prospective tuberal region (see sagittal sections, Figures 6E–I; see also Bardet et al., 2010), where *Pax6* and *Cytokeratin-8* are expressed (Figures 6D,G,J). Furthermore, we noted the onset of ADH regionalization into two domains, the future anterior and intermediate lobes (Sheng and Westphal, 1999; Rizzoti and Lovell-Badge, 2005; Reyes et al., 2008). Whereas *Tcf4* and *Pitx2* signals delineated the presumptive anterior part of the ADH (Figures 6E–G,J), *Six3* and *Raldh3* expression appears restricted to the future intermediate ADH lobe, which underlies the presumptive NH or posterior pituitary lobe (*Six3* and *Shh*-positive domain; Figures 6H–J).

These data jointly suggest that *Pitx2* is an early pre-placodal marker that intervenes during molecular regionalization of the rostral preplacodal domain into olfactory and ADH placodes. The ADH rudiment is later partitioned molecularly into two subdomains, the prospective intermediate and anterior lobes, which lie underneath the prospective NH or posterior lobe, contained for a while within the tubero-infundibular hypothalamic wall.

## Conclusion

Our analysis corroborates the median extraneural locus of the ectodermal ADH primordium at HH4, coinciding with the molecularly distinct median preplacodal domain. Data were obtained indicating that this midline locus is involved in particularly strong dorsoventral intercalative movements, oriented orthogonally to the neural/nonneural boundary. Such movements apparently represent part of the forces that quickly separate the ADH primordium from the ANR (as observed already at HH6; see Figures 3I,J), complementing other forces derived from surrounding tissues involved in head fold development, forebrain neurulation and brain and eye growth. At the end of median intercalation (e.g., HH8), the ectodermal ADH anlage appears hinged downwards under the head fold, and it already occupies a position close to its subsequent apposition to the basal hypothalamus. However, this contact only occurs somewhat later (HH12), after the initially intervening prechordal plate cells move past the median basal hypothalamus into more dorsal neighborhoods (Figure 7).

## Conflict of interest statement

The authors declare that the research was conducted in the absence of any commercial or financial relationships that could be construed as a potential conflict of interest.

## Abbreviations

abb: alar basal boundary
anp: anterior neural plate
ANR: anterior neural ridge
bp: basal plate
Di: diencephalon
dt: digestive tube
ec: ectoderm
HN: Hensen’s node
Hypoth: hypothalamus
Inf: infundibulum
mb: midbrain
N: notochord
nf: neural fold
nh: neurohypophysis
och: optic chiasm
oe: oral ectoderm
olf: olfactory placode
os: optic stalk
ov: optic vesicle
Pall: pallium
pp: prechordal plate
ppr: preplacodal region
PS: primitive streak
Pt/Th: Pretectum/Thalamus
Rh: Rhombencephalon
rm: retromamilar
rp: Rathke’s pouch
SP: secondary prosencephalon
SPall: subpallium
Tel: telencephalon.
